# Anastrozole Improves Height Outcomes in Growing Children With Congenital Adrenal Hyperplasia Due to 21-hydroxylase Deficiency

**DOI:** 10.1210/clinem/dgae771

**Published:** 2024-11-04

**Authors:** Heba Al-Rayess, Rebecca Wiersma, Lindsey Elizabeth Turner, Elise Palzer, Yesica Mercado Munoz, Kyriakie Sarafoglou

**Affiliations:** Division of Pediatric Endocrinology, University of Minnesota Medical School, Minneapolis, MN 55454, USA; Department of Pediatrics, University of Minnesota Medical School, Minneapolis, MN 55454, USA; Division of Biostatistics and Health Data Science, University of Minnesota School of Public Health, Minneapolis, MN 55455, USA; Division of Biostatistics and Health Data Science, University of Minnesota School of Public Health, Minneapolis, MN 55455, USA; Division of Pediatric Endocrinology, University of Minnesota Medical School, Minneapolis, MN 55454, USA; Division of Pediatric Endocrinology, University of Minnesota Medical School, Minneapolis, MN 55454, USA; Department of Experimental and Clinical Pharmacology, University of Minnesota College of Pharmacy, Minneapolis, MN 55455, USA

**Keywords:** congenital adrenal hyperplasia, growth, advanced bone age, aromatase inhibitors, bone mineral density, PCOS

## Abstract

**Background:**

Hyperandrogenemia resulting in estrogen-mediated accelerated bone maturation and early growth plate fusion contributes to short stature in children with congenital adrenal hyperplasia (CAH) due to 21-hydroxylase deficiency. Aromatase inhibitors block androgen conversion to estrogen and have been used off-label in children with short stature to improve adult height. There are no adequately powered studies examining the use of aromatase inhibitors in children with CAH with advanced bone age and reduced predicted adult height.

**Methods:**

Records of CAH patients treated with anastrozole were reviewed. Z-scores of bone age, predicted adult height, and height corrected for bone age were examined over an 8-year period. Outcome changes were analyzed using weighted mixed-effects models, adjusting for sex, diagnosis, age at diagnosis, and average hydrocortisone dose before and during treatment with anastrozole.

**Results:**

In 60 patients (26 females; 52 classic, 8 nonclassic) started on anastrozole therapy, the mean bone age Z-score decreased from 4.2 to 2.0 at 4 years and 1.3 at 6 years (both *P* < .001); predicted adult height Z-score improved from −2.1 to −0.45 at 4 years and 0.18 at 6 years (both *P* < .001); corrected height Z-scores improved from −1.7 to −0.33 at 4 years and 0.18 at 6 years (*P* < .001). There was no significant difference in the average total daily hydrocortisone dose used before or during treatment.

**Conclusion:**

Anastrozole decreased the rate of bone maturation and led to improved height outcomes, indicating that anastrozole could have a role as an adjunct therapy in children with CAH and advanced bone age.

Congenital adrenal hyperplasia (CAH) due to 21-hydroxylase deficiency (21OHD) results from an enzymatic defect in the cortisol synthesis pathway and shifts hormone precursors to the androgen pathway, resulting in excessive production of adrenal androgens. The negative impact that both the disease and its treatment have on adult height is well documented, with final adult heights often 1 to 2 SDs below that of the control adult population (1-3).

Hydrocortisone (HC), the glucocorticoid of choice in growing children, has a short half-life, (median elimination half-life of 58 minutes, range: 41-105 minutes), resulting in rebounding of adrenal steroids to pre-HC dose concentrations after 4 to 5 hours postdose (4, 5). As a result, children with CAH are exposed to alternating periods of hyper- and hypocortisolemia with resultant elevated adrenal androgen and, through aromatization, elevated estrogen throughout the day and early morning hours. Inadequate control of the hypothalamic-pituitary-adrenal axis can lead to accelerated growth and advanced bone age, precocious puberty, virilization in females, and formation of adrenal rests in older males. While supraphysiologic doses of HC are often used to achieve adrenal androgen and estrogen control, hypercortisolemia can also lead to impaired growth and reduced final adult height due to the inhibited release of GH and decreased activity of IGF-1 in growing bones (6).

Since estrogen is the primary hormone that regulates the fusion of the epiphysis, the use of aromatase inhibitors (AIs) has been suggested to slow epiphyseal closure and allow for a longer duration of growth by inhibiting aromatization of androgen to estrogen. Third-generation AIs have been used off-label and have been shown to be effective in treating children with short stature due to idiopathic short stature (7), constitutional delay of puberty (8, 9), disorders of puberty, and advanced bone age (10, 11) and males with GH deficiency (12). In addition to a few case reports using third-generation AIs in CAH due to 11β-hydroxylase deficiency (13-15) and 1 in 21OHD (16), there was a small study of 9 children with 21OHD treated with letrozole (17). The only larger study examined bone mineral density (BMD) and bone age maturation in 25 children with 21OHD while on treatment with anastrozole (18). Liu et al published a case series that demonstrated the efficacy of anastrozole as a monotherapy in females with nonclassic (NC) 21OHD who had bone age advancement, early pubarche, and growth acceleration but normal adrenal cortisol production (19). Our report is the first to provide longitudinal growth and bone maturation outcomes spanning up to an 8-year period, including 2 years prior and 6 years on anastrozole, in a large cohort of children with 21OHD.

## Methods

Our methods consisted of a retrospective chart review of children with 21OHD and an advanced bone age (defined as ≥2 SD). All children had a confirmed diagnosis of 21OHD based on both *CYP21A2* genetic testing and biochemical/hormonal testing including baseline and stimulated 17-hydroxyprogesterone and cortisol concentrations following a high-dose ACTH stimulation test as well as plasma renin activity, serum sodium, and potassium. Patients were treated with 1 mg daily of anastrozole in addition to their regular dose of HC, with or without fludrocortisone depending on their salt wasting status. Anastrozole was the AI used for the study because it has been shown to effectively slow down bone age acceleration and increase height potential with less impact on testosterone concentrations compared to letrozole (20). Treatment with anastrozole was discontinued when the patient's bone age reached 14 years in girls and 16 years in boys. Data were collected and managed using Research Electronic Data Capture tools hosted at the University of Minnesota, a secure, Health Insurance Portability and Accountability Act-protected, web-based software platform. The study was approved by the University of Minnesota institutional review board. Clinical data including bone age, bone age Z-scores, height, height Z-scores, pubertal status, and HC dose were analyzed for visits starting 2 years prior to treatment with anastrozole up until 6 years on treatment. HC dosing was adjusted to maintain 17-hydroxyprogesterone concentrations below 1000 ng/dL and androstenedione concentrations within the range for age and Tanner stage of development. Puberty onset was determined by breast development at Tanner stage 2 or more in girls and testicular size ≥4 mL in boys. Predicted adult height (PAH) was calculated using height and bone age at each visit based on Greulich and Pyle method (21). PAH Z-score was calculated using the Centers for Disease Control and Prevention (CDC) Growth Chart SAS Macro by entering the PAH for height and the chronological age of 19 years and 11 months (oldest age for adults on the CDC growth charts) (22). PAH Z-scores can be only calculated for children with a bone age of 7 years or older (21). Height Z-scores were adjusted for bone age as children with CAH and advanced bone age can be misclassified as tall compared to children of the same age and sex. Height Z-scores adjusted for bone age were calculated using the CDC growth chart SAS Macro by using the height at the visit and replacing chronological age with the patient's bone age obtained at the same time as the height measurement.

Dual X-ray absorptiometry (DXA) scans were performed to evaluate BMD during treatment with anastrozole using GE Lunar Prodigy (GE Healthcare). Total body BMD (TBMD) Z-scores and L2-L4 Z-scores were extracted from the DXA scan using encore™ software (platform version 13.6; GE Healthcare). TBMD Z-scores and L2-L4 Z-scores were adjusted for height-for-age Z-scores (TBMD_HAZ_ and L2-L4_HAZ_) based on the method described by Zemel et al (23).

### Statistical Analysis

Descriptive statistics for our study population were summarized by n (percent) or mean (SD) at various time points on anastrozole. A patient was considered on a GnRH analogue at a given time point if they were on a GnRH analogue at any point during the specified time period. Average HC dose was defined by the average daily HC dose throughout the specified time period and is expressed in mg/m^2^/day.

All data were measured during clinic visits, which occured at unsystematic times for each child. Therefore, to analyze outcomes at specific time points, standardized Z-score measures (for bone age, height, height adjusted for bone age, weight, PAH, and total body and lumbar BMD) and unstandardized PAH were estimated for each individual and time point by fitting a subject-specific Gaussian kernel smoother with an adaptively chosen bandwidth (24) using the “stats” package in R (25). Descriptive statistics for each estimated patient outcome were also calculated.

Weighted mixed-effects models were used to analyze the effect of treatment on the change in Z-score measures and PAH at specific time points. The impact of GnRH analogues on change in bone age Z-score, height adjusted for bone age Z-score, and PAH Z-score over the course of anastrozole treatment was assessed by weighted linear regression models adjusted for age at diagnosis, diagnosis, sex, time on anastrozole, average HC dose before starting anastrozole, and average HC dose while taking anastrozole. Weights were calculated by taking the inverse of the distance in days between the start of anastrozole and its nearest Z-score measurement, thus giving more weight to Z-scores measured closer to the start of treatment and lower weight to Z-scores measured further from the start of treatment. The end of anastrozole treatment for each individual was defined as the last visit at which the Z-score or desired outcome was measured before stopping treatment.

For TBMD and L2-L4 Z-scores, change in Z-score over treatment was analyzed by a mixed-effects model with a random intercept for subject and fixed effects for height Z-score and a treatment indicator. The impact of GnRH analogues on BMD Z-scores was also assessed by mixed-effects modeling as described earlier with an additional fixed effect for GnRH analogue use at any point during anastrozole. All statistical analyses were conducted in R version 4.3.1.

## Results

### Patient Characteristics

The characteristics of the 60 patients diagnosed with CAH are listed in Table 1. Although the dataset has 60 patients, we do not have data for all 60 patients at any time point. These patients represent all patients at our institution who have been on anastrozole therapy from the year 2003 to the time of submission of this manuscript. The average age at diagnosis was 2.1 years, although it was later in children with NC CAH (6.4 years). Out of the 60 children with CAH, 6 had NC–CAH by genotype but a clinical phenotype consistent with intermediate between NC and salt virilizing clinical phenotype, as all 6 had suboptimal cortisol secretion in response to high-dose ACTH stimulation test and signs of virilization (penile or clitoral enlargement) and advanced bone age. The average age at initiation of anastrozole was 7.7 (2.4) years, and treatment lasted for an average of 4.9 (2.6) years. There are 13 patients who are still on anastrozole therapy at the time of submission of this manuscript. A total of 45 (75.0%) children were prepubertal at the time of initiation of anastrozole therapy. The average age of puberty onset in this cohort was 10.1 years (SD 2.6). For boys, puberty started at an average age of 10.9 years (SD 2.6, median 11.3, range 4.4 to 15.0). For girls, the average onset of puberty was 9.1 (SD 2.3, median 9.1, range 4.9 to 13.2).

**Table 1.** Patient characteristics at the beginning of treatment and at 1 year, 2 years, 4 years, and 6 years

	Years on aromatase				
Covariate	0	1	2	4	6
Total	60	57	53	32	19
Sex (%) Male	34 (56.7)	31 (54.4)	29 (54.7)	19 (59.4)	12 (63.2)
Female	26 (43.3)	26 (45.6)	24 (45.3)	13 (40.6)	7 (36.8)
Diagnosis (%)					
Salt wasting	32 (53.3)	29 (50.9)	26 (49.1)	14 (43.8)	7 (36.8)
Simple virilizing	20 (33.3)	20 (35.1)	19 (35.8)	13 (40.6)	10 (52.6)
Nonclassic	8 (13.3)	8 (14.0)	8 (15.1)	5 (15.6)	2 (10.5)
Age, years (SD)	7.7 (2.4)	8.7 (2.5)	9.7 (2.5)	10.8 (2.1)	11.9 (1.9)
Male	8.0 (2.6)	9.0 (2.7)	10.1 (2.7)	11.0 (2.4)	12.0 (2.1)
Female	7.3 (2.2)	8.3 (2.2)	9.1 (2.2)	10.4 (1.6)	11.8 (1.7)
Salt wasting	8.2 (2.1)	9.2 (2.2)	10.2 (2.2)	11.7 (1.7)	13.1 (1.4)
Simple virilizing	6.8 (3.0)	7.8 (3.0)	8.7 (3.0)	9.4 (2.0)	10.8 (1.7)
Nonclassic	8.0 (1.6)	9.0 (1.6)	10.0 (1.6)	12.1 (1.5)	13.7 (0.65)
Age at diagnosis, years (SD)	2.1 (3.1)	2.3 (3.1)	2.4 (3.2)	2.7 (3.2)	2.3 (2.9)
Male	2.5 (3.5)	2.8 (3.5)	3.0 (3.6)	2.9 (3.5)	2.3 (2.8)
Female	1.7 (2.5)	1.7 (2.5)	1.8 (2.5)	2.6 (3.0)	2.4 (3.1)
Salt wasting*^a^*	0.32 (1.6)	0.35 (1.7)	0.39 (1.8)	0.04 (0.11)	0.01 (0.01)
Simple virilizing*^b^*	3.3 (2.9)	3.3 (2.9)	3.5 (3.0)	3.6 (2.7)	3.0 (2.7)
Nonclassic*^c^*	6.4 (1.9)	6.4 (1.9)	6.4 (1.9)	7.4 (1.4)	6.8 (0.09)
Pubertal status (%) Prepubertal	46 (76.7)	37 (64.9)	37 (69.8)	19 (59.4)	10 (52.6)
Pubertal	14 (23.3)	20 (35.1)	16 (30.2)	13 (40.6)	9 (47.4)
GnRH analogue () No	54 (90.0)	43 (75.4)	32 (60.4)	18 (56.2)	6 (31.6)
Yes	6 (10.0)	14 (24.6)	21 (39.6)	14 (43.8)	13 (68.4)

^*a*^The median age at diagnosis was 0.01 years.

^*b*^The median age at diagnosis was 2 years.

^*c*^The median age at diagnosis was 6.6 years.

Thirty-two (53.3%) children received pubertal suppression with a GnRH agonist at some point over the course of the study time period. The average age of GnRH agonist initiation for boys was 8.3 years (SD 2.5, median 9.5, range 2.8 to 11.5). For girls GnRH agonists were started at an average age of 7.7 years (SD 1.6, median 7.8, range 5.0 to 9.9).

### Patient Outcomes—Bone Age

**Figure 1.**
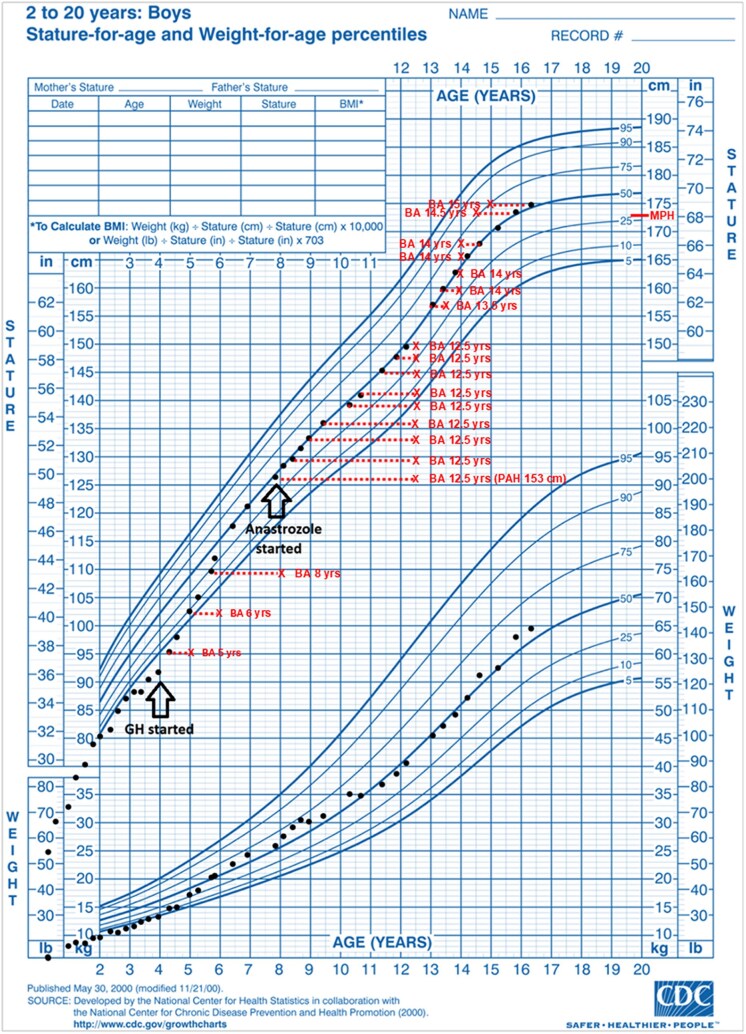
Height and weight growth chart for a male patient from our cohort with salt-wasting SW CAH treated with hydrocortisone and fludrocortisone since infancy. He was treated with GH for idiopathic short stature at age 4 years. He was lost to follow-up from ages 5 to 8 years. Upon his return at age 8, his bone age was advanced at 12.5 years with a predicted adult height of 153 cm (midparental target height 174 cm), and he was started on anastrozole therapy. From ages 8 to 13, his bone age advanced just 1 year to 13.5 years. He remained on anastrozole until age 16.4 years (bone age 15 years) with a near adult height of 175 cm. The “X” indicates bone age at the corresponding chronological age. The dotted line shows the gap between chronological age and bone age. Abbreviations: CAH, congenital adrenal hyperplasia; MPH, midparental height; PAH, predicted adult height.

The mean bone age Z-score was 4.2 at the time of initiation of anastrozole therapy. Mean bone age Z-scores were determined at 1, 2, 4, and 6 years on anastrozole therapy. Bone age Z-score decreased at each subsequent measured time point on anastrozole therapy, and a longer time on anastrozole therapy was associated with greater reductions in bone age Z-score (Tables 2 and 3; *P* < .001). This relationship was also seen within each sex category. The HC dose remained within the recommended range per CAH consensus guidelines during the anastrozole treatment period (26). The mixed-effects model that was used to determine the effect of anastrozole over the course of treatment found that the effect size increased with longer time on AI, with a mean decrease in bone age Z-score of −3.99 [95% confidence interval (CI) −4.84, −3.14; *P* < .001] in subjects who received 6 years of anastrozole therapy. Anastrozole also significantly decreased bone age Z-scores across each consecutive treatment interval, with significant decreases in mean bone age Z-scores seen at 0 to 2 years (−1.14, 95% CI −1.49, −0.80; *P* < .001), 2 to 4 years (−1.60, 95% CI −1.94, −1.26; *P* < .001), and 4 to 6 years (−1.38, 95% CI −1.74, −1.02; *P* < .001). An example of anastrozole's effect of slowing bone age maturation can be seen in Fig. 1 in which the bone age of a boy advanced just 1 year from 8 to 13 years of age.

**Table 2.** Bone age and growth outcome measures 2 years prior and over 6 years of anastrozole therapy

	Years on anastrozole (SD)					
Covariate	2 prior	0	1	2	4	6
Bone age Z-score	2.0 (1.2)	4.2 (2.5)	3.7 (2.5)	3.0 (2.3)	2.0 (2.0)	1.3 (1.7)
Male	1.8 (1.3)	4.6 (2.7)	4.2 (2.5)	3.2 (2.4)	2.4 (2.1)	1.9 (1.8)
Female	2.2 (1.1)	3.7 (2.2)	3.2 (2.3)	2.7 (2.2)	1.4 (1.8)	0.41 (1.1)
SW	1.8 (1.1)	3.5 (2.0)	3.1 (2.3)	2.6 (2.2)	1.4 (1.8)	0.62 (1.2)
SV	2.5 (1.5)	5.6 (2.9)	4.9 (2.5)	4.1 (2.2)	3.4 (1.6)	2.3 (1.5)
NC	2.9 (NA)	3.1 (1.9)	2.8 (1.9)	1.7 (1.6)	0.07 (0.9)	−0.93 (0.16)
PAH (cm)	160 (11.7)	156 (10.4)	159 (10.7)	162 (10.7)	168 (10.3)	173 (10.6)
Male	169 (6.5)	162 (8.6)	164 (9.2)	168 (9.2)	174 (7.1)	179 (5.8)
Female	149 (4.7)	149 (7.6)	152 (8.2)	155 (8.0)	160 (8.7)	162 (8.7)
SW	160 (11.6)	157 (10.3)	158 (11.0)	161 (11.5)	166 (9.4)	173 (9.9)
SV	164 (13.3)	155 (11.4)	159 (11.8)	162 (11.3)	169 (12.6)	173 (12.3)
NC	147 (NA)	155 (8.6)	159 (7.5)	163 (6.4)	173 (4.7)	177 (NA)
PAH Z-score	−1.6 (0.99)	−2.1 (1.2)	−1.7 (1.2)	−1.3 (1.2)	−0.45 (1.1)	0.18 (1.0)
Male	−1.1 (0.91)	−2.1 (1.2)	−1.7 (1.3)	−1.2 (1.3)	−0.4 (0.99)	0.35 (0.82)
Female	−2.2 (0.71)	−2.2 (1.2)	−1.8 (1.2)	−1.3 (1.2)	−0.51 (1.3)	−0.12 (1.3)
SW	−1.7 (0.97)	−2.0 (1.1)	−1.8 (1.2)	−1.4 (1.3)	−0.83 (0.98)	0.03 (0.95)
SV	−0.97 (1.0)	−2.2 (1.4)	−1.8 (1.3)	−1.4 (1.2)	−0.45 (1.2)	0.09 (0.95)
NC	−2.4 (NA)	−2.0 (1.0)	−1.3 (1.2)	−0.65 (1.1)	0.6 (0.65)	2.1 (NA)
PAH Z-score minus target height Z-score	−1.4 (1.2)	−2.3 (1.2)	−2.0 (1.3)	−1.5 (1.2)	−0.64 (1.2)	−0.15 (0.73)
Male	−0.79 (1.2)	−2.2 (1.4)	−2.0 (1.4)	−1.5 (1.5)	−0.55 (1.2)	−0.01 (0.44)
Female	−2.1 (0.87)	−2.4 (1.1)	−2.0 (1.1)	−1.5 (1.0)	−0.78 (1.2)	−0.38 (1.1)
SW	−1.4 (1.2)	−2.1 (1.2)	−1.8 (1.4)	−1.4 (1.4)	−0.75 (1.5)	−0.31 (0.86)
SV	−1.2 (1.3)	−2.5 (1.3)	−2.2 (1.2)	−1.7 (1.1)	−0.81 (1.0)	−0.21 (0.46)
NC	NA (NA)	−2.6 (1.1)	−2.0 (1.1)	−1.4 (0.86)	0.08 (0.49)	1.4 (NA)
Height Z-score corrected for bone age	−1.0 (0.91)	−1.7 (1.0)	−1.4 (1.1)	−1.0 (1.0)	−0.33 (0.89)	0.18 (0.76)
Male	−0.89 (0.94)	−1.8 (1.0)	−1.5 (1.1)	−1.1 (1.1)	−0.4 (0.81)	0.23 (0.66)
Female	−1.1 (0.87)	−1.6 (0.99)	−1.2 (1.0)	−0.85 (0.94)	−0.24 (1.0)	0.09 (0.99)
SW	−1.1 (0.91)	−1.7 (0.96)	−1.4 (0.98)	−1.1 (1.0)	−0.66 (0.77)	0.02 (0.77)
SV	−0.71 (0.94)	−1.8 (1.2)	−1.5 (1.2)	−1.1 (1.0)	−0.35 (0.86)	0.15 (0.66)
NC	−1.7 (NA)	−1.6 (0.88)	−0.86 (1.1)	−0.34 (1.0)	0.61 (0.71)	1.6 (NA)
Average HC dose, mg/m^2^/day	10.5 (2.8)	12.1 (3.4)	12.2 (2.4)	11.9 (2.5)	12.2 (2.9)	12.8 (2.6)
Male	10.6 (1.8)	11.7 (2.8)	12.4 (2.1)	12.0 (2.4)	11.8 (3.0)	12.6 (2.2)
Female	10.3 (3.8)	12.5 (4.0)	12.0 (2.7)	11.7 (2.8)	12.9 (2.9)	13.1 (3.4)
SW	10.2 (2.7)	11.4 (2.7)	12.0 (2.2)	12.1 (2.6)	13.0 (3.0)	13.2 (2.5)
SV	11.6 (2.9)	13.8 (4.1)	13.1 (2.3)	12.3 (2.5)	12.2 (2.8)	13.3 (2.4)
NC	7.3 (NA)	9.8 (1.9)	10.3 (2.3)	10.1 (1.7)	9.8 (2.3)	9.1 (1.6)

Abbreviations: HC, hydrocortisone; NA, not available; NC, nonclassic; PAH, predicted adult height; SV, salt virilizing; SW, salt wasting.

**Table 3.** Weighted mixed effects model for the main bone age and growth outcomes

Outcome	0-1 year (95% CI)	0-2 years (95% CI)	2-4 years (95% CI)	0-4 years (95% CI)	4-6 years (95% CI)	0-6 years (95% CI)
Bone age Z-score	−0.5 (−0.75, −0.25); *P* < 0.001	−1.14 (−1.49, −0.8); *P* < 0.001	−1.6 (−1.94, −1.26); *P* < 0.001	−2.73 (−3.39, −2.07); *P* < 0.001	−1.38 (−1.74, −1.02); *P* < 0.001	−3.99 (−4.84, −3.14); *P* < 0.001
PAH	2.08 (0.96, 3.19); *P* < 0.001	5.19 (3.7, 6.68); *P* < 0.001	7.81 (6.36, 9.26); *P* < 0.001	12.31 (9.49, 15.14); *P* < 0.001	6.31 (4.64, 7.98); *P* < 0.001	19.46 (15.59, 23.33); *P* < 0.001
PAH Z-score	0.3 (0.14, 0.47); *P* < 0.001	0.75 (0.53, 0.97); *P* < 0.001	1.12 (0.92, 1.32); *P* < 0.001	1.76 (1.35, 2.17); *P* < 0.001	0.91 (0.67, 1.15); *P* < 0.001	2.77 (2.22, 3.32); *P* < 0.001
Height Z-score corrected for bone age	0.27 (0.16, 0.38); *P* < 0.001	0.61 (0.45, 0.77); *P* < 0.001	0.9 (0.73, 1.07); *P* < .001	1.48 (1.17, 1.79); *P* < .001	.76 (0.58, 0.93); *P* < .001	2.36 (1.93, 2.8); *P* < .001

These models are all adjusted for age at diagnosis, diagnosis, sex, average hydrocortisone dose before starting aromatase, and average hydrocortisone dose during the desired time period.

Abbreviations: CI, confidence interval; PAH, predicted adult height.

### Patient Outcomes—Predicted Linear Growth

Mean PAH Z-scores were determined at 0, 1, 2, 4, and 6 years on anastrozole therapy. The PAH Z-score increased at each subsequent time point on anastrozole therapy, and a longer time on anastrozole therapy was associated with a greater increase in the PAH Z-score (Table 3). The overall mean PAH Z-score increased from −2.1 at the start of therapy to 0.18 at 6 years of treatment (*P* < .001). This effect was consistent when stratified by sex, with increases from −2.1 to 0.35 and −2.2 to −0.12 seen in male and female patients, respectively. Similarly, the mixed-effects model showed that higher values of both PAH and PAH Z-score are significantly associated with a longer time on anastrozole. In the first year of anastrozole therapy, there was a mean increase in PAH of 2.08 cm and an increase in Z-score of 0.3, while after 6 years of anastrozole therapy there was a mean PAH increase of 19.46 cm corresponding to a mean Z-score increase of 2.77 (both *P* < .001). Anastrozole treatment was also significantly associated with an increased PAH and PAH Z-score across each consecutive treatment interval, with increases seen at 0 to 2 years, 2 to 4 years, and 4 to 6 years. This effect remained significant when height Z-scores were corrected for bone age. Height Z-scores adjusted for bone age were also shown to progressively increase over the treatment duration from −1.7 to 0.18 at 6 years of treatment. This effect was significant when adjusted height Z-scores were compared from the beginning up to 6 years of treatment as well as when compared at 2-year intervals.

### Effect of GnRH Analogues

There were no significant differences observed in the effect of AIs on decreasing bone age Z-scores over time based on whether or not patients received GnRH analogue (Table 4).

**Table 4.** Weighted linear model looking at the effect of GnRH analogues on the change in the outcomes from start to end of anastrozole

Outcome of interest	GnRH analogues (95% CI)
Change in bone age Z-score from start to end of anastrozole	−1.22, (−3.48, 1.03); *P* = .28
Change in predicted adult height Z-score from start to end of anastrozole	0.4, (−4.55, 5.34); *P* = .83
Change in height Z-score corrected for bone age from start to end of anastrozole	−0.46, (−1.83, 0.91); *P* = .5

These models were adjusted for age at diagnosis, diagnosis, sex, time on anastrozole, average hydrocortisone dose before starting anastrozole, and average hydrocortisone dose while taking anastrozole.

Abbreviations: CI, confidence interval.

### Monitoring Labs and Imaging Studies

Out of the 26 females, 21 had at least 1 ovarian ultrasound during treatment. Females typically had 1 to 2 pelvic ultrasounds during treatment with anastrozole. One pubertal female (age 12.7 years) had an ultrasound while on treatment with anastrozole for 2.8 years that demonstrated a simple right ovarian cyst. She had fenestration of the simple ovarian cyst laparoscopically. Her pelvic ultrasound prior to the procedure showed the left ovary to be of normal size with no cysts or follicles, suggesting that anastrozole-driven LH overdrive was not the reason for the cyst. She did not restart anastrozole at that point as most of her growth had been completed.

All patients on anastrozole had their liver function tests measured while on treatment 2 to 3 times a year during their clinic follow-up visits for 21OHD. One patient was noted to have an elevation of liver enzymes during a hospitalization for vomiting and diarrhea. He was diagnosed with presumed viral gastroenteritis, and liver function tests normalized following resolution of his acute illness. Two other patients developed persistently elevated liver enzymes while on anastrozole, both with abdominal ultrasound findings of hepatic steatosis most suggestive of metabolic dysfunction associated with steatotic liver disease (MASLD) in the setting of obesity (body mass index >99th percentile for age). Other etiologies causing elevated liver enzymes were excluded clinically and/or through laboratory testing. Anastrozole was temporarily discontinued in 1 of the 2 patients and resumed once the diagnosis of MASLD was made. Liver enzymes normalized and remained normal following reinitiation of anastrozole, supporting the idea that this transient elevation was not a side effect of anastrozole. The other patient had normalization of liver enzymes while remaining on anastrozole. He was also evaluated by pediatric gastroenterology who concluded that the transient elevation of his alanine aminotransferase was possibly triggered by a viral infection, though he remains at risk for MASLD.

Fifty-two (87%) patients had at least 1 DXA scan during the treatment period with anastrozole. There was no significant change in the total body or lumbar BMD Z-scores over the course of treatment (Table 5; *P* = .12, .44 respectively). When adjusting BMD Z-scores for height Z-scores based on previously published equations by Zemel (23), there was no significant change in the lumbar BMD Z-scores, but there was an increase in the total body BMD Z-scores by an average of 0.44 (*P* = .04). The use of GnRH analogues did not significantly affect the change in BMD Z-scores over the course of anastrozole treatment (Table 6). Patients and their parents did not report an increase in clinical symptoms associated with androgen excess such as hair growth, acne, or increased virilization.

**Table 5.** Bone mineral density Z-scores comparison at the beginning and end of treatment with anastrozole with analysis of their change over time

	Mean value at beginning of treatment*^a^*	Mean value at end of treatment*^a^*	BMD change over duration of treatment (95% CI)*^b^*	*P*-value
Total body BMD z-score	0.95 (1.02), n = 24	0.87 (1.27), n = 19	0.31 (−0.08, 0.7)	*P* = .12
Lumbar Z-score	0.56 (1.12), n = 27	0.44 (1.05), n = 20	−0.12 (−0.41, 0.18)	*P* = .44
Zemel adjusted BMD Z-score	0.07 (0.88), n = 24	0.83 (1.01), n = 19	0.44 (0.03, 0.86)	*P* = .04
Zemel adjusted lumbar Z-score	−0.13 (1.08), n = 27	0.33 (0.93), n = 20	−0.07 (−0.39, 0.26)	*P* = .68

Abbreviations: BMD, bone mineral density.

^*a*^These values are estimated only for patients who have a scan within 1 month of initiation or termination of treatment or who have a scan before and after initiation or termination.

^*b*^These come from a mixed effects model with a random intercept for subject and fixed effects for time on treatment (start or end) and height for age Z-score. These analyses only have 7 patients with both scans at the start and end of treatment for BMD Z-score and 9 patients with both scans at the start and end of treatment for lumbar Z-scores.

**Table 6.** Linear mixed effects model for the effect of GnRH analogue on change of BMD Z-scores over the course of anastrozole treatment

Outcome of interest	GnRH analogues (95% CI)	*P*-value
BMD Z-score	−0.17 (−0.86, 0.53)	.64
Lumbar Z-score	−0.22 (−0.98, 0.53)	.56
Zemel adjusted BMD Z-score with height Z-score	−0.17 (−0.9, 0.57)	.66
Zemel adjusted lumbar Z-score	−0.22 (−0.98, 0.55)	.58

These models were adjusted for time on treatment (indicator with start or end) and height for age Z-score.

Abbreviations: BMD, bone mineral density.

## Discussion

We present the first longitudinal study that examines the effect of anastrozole as an adjunctive therapy to target excess adrenal androgens in growing patients with CAH. Our study found a significant and progressive decrease in bone age Z-scores during anastrozole treatment, which resulted in a significant gain in growth potential, as demonstrated by an average 13-cm increase in the predicted adult height in girls and 17 cm in boys over the 6 years of treatment. This effect of decreased skeletal maturation rate was achieved without the need for increased doses of HC and without the development of clinical symptoms associated with androgen excess.

Studies that have reported the use of AIs in other disorders of puberty and growth are summarized in Table 7 (8, 9, 11, 12, 27, 28). The overall results of these studies indicate that adding an AI to other growth-promoting treatments universally improves the near adult height or PAH in idiopathic short stature, growth hormone deficiency, constitutional delay of growth and puberty, and central precocious puberty. AI monotherapy also improved PAH in idiopathic short stature and constitutional delay of growth and puberty. Compared to PAH/near adult height gains in these studies, our cohort had greater height gains than most prior studies. This is likely explained by the higher degree of bone age advancement related to the pathophysiology of CAH, the longer duration of treatment, and the earlier age at initiation of treatment in our study.

**Table 7.** Comparison of studies using aromatase inhibitors for growth promotion

Author (year)	Study design	Diagnosis	Comparators	Mean age at AI start (yrs)*^a^*	Mean bone age at AI start (yrs)*^a^*	Pubertal status*^a^*	Treatment duration	PAH gain (cm)*^a^*	NAH gain (cm)*^a^*
Hero et al (2005)	RCT	ISS (males)	Letrozole Placebo	11.0 ± 1.7	9.1 ± 2.3	Tanner 1-3	24 months	5.9 NSC	n/a
Mauras et al (2016)	Randomized trial	ISS (males)	AI (anastrozole/letrozole) GH GH/AI	14.1 ± 0.1	12.7 ± 0.2	Tanner 2-3	24-36 months	n/a	18.2*^b^* 20.6 22.5
Rothenbuhler et al (2015)	Randomized pilot trial	ISS (males)	GH GH + anastrozole	15.2 ± 1.2	14.5 ± 0.8	Testicular volume 22.2 ± 5	Mean 19 months	n/a	5.9 ± 4.5*^c^* 10.5 ± 5.2
Wickman et al (2001)	RCT	Delayed puberty (males)	testosterone + letrozole Testosterone + placebo No treatment	15.2 ± 0.8	13.1 ± 0.6	Testicular volume 5.5 ± 1.9	18 months	5.1 NSC NSC	n/a
Mauras et al (2008)	RCT	GHD (males)	GH + anastrozole GH + placebo	13.8 ± 0.3	13.7 ± 0.2	Tanner 2-4	12-36 months	4.5, 6.7 at 24, 36 months 1, 1 at 24, 36 months	n/a
Papadimitriou et al (2016)	Prospective	CPP (females)	Lupron + anastrozole Lupron	8.91 ± 0.98	BAA 1.88 ± 1.11	Tanner 2 or above	24 months	7.51 1.92	n/a
Salehpour et al (2010)	RCT	CDGP (males)	Letrozole Oxandrolone Placebo	12.6-14.6	12.18 ± 1.1	Tanner 1	2 years	6.10 ± 1.9 1.9 ± 1.0 1.4 ± 0.80	n/a
Current study (2024)	Retrospective	CAH (males, females)	Anastrozole/HC HC	7.7 (±2.5)	10.8 (±2.2)	23.2% Tanner 2 or above	6 years	Males 17 cm Females 13 cm	n/a

Abbreviations: AI, aromatase inhibitors; BAA, bone age advancement; CAH, congenital adrenal hyperplasia; CDGP, constitutional delay of growth and puberty; CPP, central precocious puberty; GHD, growth hormone deficiency; HC, hydrocortisone; ISS, idiopathic short stature; NAH, near adult height; NSC, no significant change; PAH, predicted adult height; RCT, randomized controlled trial.

^*a*^For treatment group.

^*b*^NAH gain at 24 months compared to baseline.

^*c*^PAH was based on an equation rather than bone age in this study.

Accelerated bone maturation in children with CAH is mainly caused by aromatization of adrenal androgens but can also be exacerbated by central precocious puberty. Due to the short half-life of HC, children with CAH are exposed to chronic, intermittent elevations in adrenal androgen and estrogen that occur in between doses and in the early morning hours when typically the evening HC dose has already washed out and the adrenals are maximally stimulated following the morning ACTH surge (4, 5). Excess adrenal sex steroids can prime the hypothalamic-pituitary-gonadal axis and result in central precocious puberty, which can further contribute to bone age advancement and a compromised final adult height. Therefore, it is not unusual for children with CAH to enter puberty early due to chronic exposure to elevated adrenal sex steroids requiring treatment with GnRH analogues, especially if their bone age at the time of puberty onset is advanced, predicting a reduced adult height. When adjusting for GnRH analogue treatment in our cohort, anastrozole was found to have an independently significant effect in decreasing bone age Z-scores and improving PAH regardless of puberty suppression status. This reflects the fact that these treatments address 2 different pathophysiologic mechanisms: GnRH analogues suppress central puberty by downregulating pituitary GnRH receptors and ultimately decreasing ovarian or testicular sex hormone production, while anastrozole blocks the aromatization of the excessive adrenal androgens to estrogens.

Our study, along with multiple prior studies, found no association between AIs and decreased bone density (11, 18, 19, 27). It has been hypothesized that the use of AIs in children could potentially decrease BMD because of estrogen's role in bone accrual and turnover through stimulation of osteoblast activity and inhibition of osteoclast activity and differentiation (29). Halper et al speculated that increased adrenal androgens may compensate for the effect of decreased estrogen levels on bone (18). In addition, both androgen and estrogen can stimulate osteoblastic activity, counteract osteoblast apoptosis, and stimulate osteoclast apoptosis (29, 30). Vertebral abnormalities were observed during treatment with letrozole in boys with idiopathic short stature or delayed puberty (7). However, a subsequent report by the same authors demonstrated improvement in these abnormalities during follow-up (31).

Females with CAH are at risk for a secondary mimic of polycystic ovarian syndrome (PCOS). This is likely due to both the effects of elevated adrenal androgens in utero and intermittently chronically elevated androgen while on therapy. Diagnosis of PCOS during the adolescent years is challenging due to the overlap of normal pubertal physiological changes (irregular menstrual cycles, acne, and polycystic ovarian morphology on pelvic ultrasound) with adult PCOS diagnostic criteria, which require the exclusion of NC 21OHD (32). Current guidelines recommend avoiding the use of pelvic ultrasound in the diagnosis of PCOS in females until at least 8 years postmenarche. As most female CAH patients in our report have not reached the 8-year cut-off for repeat ovarian ultrasound, future follow-up study to evaluate for PCOS is warranted.

There have been limited case reports describing anastrozole-induced hepatitis in women undergoing treatment with anastrozole for breast cancer, although the mechanism of this relationship remains uncertain (33-36). One patient in our cohort developed elevated liver enzymes in the setting of acute viral illness, which resolved following resolution of acute illness. Two obese children, 1 male and 1 female, had abnormal liver enzymes in the setting of MASLD. No other patients showed abnormal liver function testing during treatment with anastrozole.

While our study showed that anastrozole effectively optimizes growth in patients with CAH via inhibition of estrogen-mediated bone age advancement, it does not target other androgen or ACTH-mediated side effects, such as increased virilization and testicular adrenal rests. Emerging nonsteroidal therapies, including CRF1 receptor antagonists, have the potential to decrease ACTH-driven adrenal androgen excess and its associated comorbidities during childhood even before clinical symptoms develop in patients with CAH (37-42).

Our study evaluated “real-world” use of AIs outside the confines of a clinical trial in a retrospective, observational database. Therefore, it does not include patients with CAH and advanced bone age treated with HC alone, as the majority of families opted for anastrozole therapy. Patient visits occurred at varying frequencies, and data collected at these visits were not consistent. Additionally, only a subset of the patients was on anastrozole therapy for 6 or more years and may not be representative of all CAH patients. The addition of AIs was briefly discussed in the most recent clinical practice guidelines for CAH, with the authors citing the need for further studies demonstrating their safety and efficacy (26).

Based on our results, anastrozole can be an effective adjunctive therapy in children with CAH and advanced bone age to optimize their growth. Nonetheless, a longitudinal randomized controlled clinical trial is needed to assess long-term outcomes. Although no significant side effects were observed in our cohort, we suggest monitoring liver function tests and BMD before starting AIs and periodically during treatment. As 1 out of 26 (3.8%) female patients developed an ovarian cyst, which is a lower frequency than in unaffected females aged 5 to 18 years of age (13.1%) (43), we recommend a periodic ovarian ultrasound when using anastrozole.

## Data Availability

Restrictions apply to the availability of data generated or analyzed during this study to preserve patient confidentiality. The corresponding author will, on request, detail the restrictions and any conditions under which access to some data may be provided.
